# Non-Oncologic Applications of Nanomedicine-Based Photo-Therapy

**DOI:** 10.3390/biomedicines9020113

**Published:** 2021-01-25

**Authors:** Su Woong Yoo, Gyungseok Oh, Jin Chul Ahn, Euiheon Chung

**Affiliations:** 1 Department of Nuclear Medicine, Chonnam National University Hwasun Hospital, Jeollanam-do 58128, Korea; yoosw.md@gmail.com; 2 Department of Biomedical Science and Engineering, Gwangju Institute of Science and Technology (GIST), Gwangju 61005, Korea; gsoh87@gmail.com; 3 Medical Laser Research Center and Department of Biomedical Science, Dankook University, Cheonan 31116, Korea; jcahn@dankook.ac.kr; 4 Department of Physics and Photon Science, Gwangju Institute of Science and Technology (GIST), Gwangju 61005, Korea; 5 AI Graduate School, Gwangju Institute of Science and Technology (GIST), Gwangju 61005, Korea

**Keywords:** photosensitizers, phototherapy, photodynamic therapy, photothermal therapy, non-oncologic applications

## Abstract

Phototherapy is widely applied to various human diseases. Nanomedicine-based phototherapy can be classified into photodynamic therapy (PDT) and photothermal therapy (PTT). Activated photosensitizer kills the target cells by generating radicals or reactive oxygen species in PDT while generating heat in PTT. Both PDT and PTT have been employed for treating various diseases, from preclinical to randomized controlled clinical trials. However, there are still hurdles to overcome before entering clinical practice. This review provides an overview of nanomedicine-based phototherapy, especially in non-oncologic diseases. Multiple clinical trials were undertaken to prove the therapeutic efficacy of PDT in dermatologic, ophthalmologic, cardiovascular, and dental diseases. Preclinical studies showed the feasibility of PDT in neurologic, gastrointestinal, respiratory, and musculoskeletal diseases. A few clinical studies of PTT were tried in atherosclerosis and dry eye syndrome. Although most studies have shown promising results, there have been limitations in specificity, targeting efficiency, and tissue penetration using phototherapy. Recently, nanomaterials have shown promising results to overcome these limitations. With advanced technology, nanomedicine-based phototherapy holds great potential for broader clinical practice.

## 1. Introduction

Phototherapy is the use of light to treat disease. Although sunlight was used to treat disease (Heliotherapy) from BC 1400s, phototherapy′s scientific documentation could be found at the end of the 19th century [1]. In 1893, Niels Finsen, a dermatologist in Denmark, treated lupus vulgaris by filtered sunlight [2], for which he obtained Finsen the Nobel Prize in 1903. For the past several decades, phototherapy has been widely applied to various clinical diseases with nanomedicine′s advancement, such as new generation photosensitizers (PSs) [3,4].

The current phototherapy with exogenous PSs can be broadly classified into photodynamic therapy (PDT) and photothermal therapy (PTT) [5,6,7]. In PDT, PSs generate cytotoxic chemical agents under photoactivation by light. On the other hand, PSs can produce overheating under light irradiation in PTT. The PTT agents are employed to achieve the selective heating of the target tissue. As a result of both phototherapies, the activated PSs promote apoptotic and necrotic cell death in the target lesion via distinct mechanisms [8,9].

PDT was used to localize lesions and assess therapeutic effects with hematoporphyrin derivatives in the 1960s [10,11]. After the first clinical approval of porfimer sodium (Photofrin; Concordia Laboratories Inc, St Michael, Barbados) in 1993, PDT has become the most site-specific remedy applicable to the treatment of oncological disease. The PSs have several shortcomings that present challenges to their wide applications. For example, highly conjugated organic PSs, including porphyrin and chlorin derivatives, are difficult to dissolve and present serious aggregation tendencies leading to unfavorable bioavailability and biodistribution [12]. The lack of selectivity for lesion sites can lead to off-target side effects, such as hepatic spots and lytic necrosis. Moreover, uncontrollable photoactivity and slow clearance could trigger post-treatment hazards [13].

PTT has recently emerged as an important and efficient strategy for cancer treatment with its short treatment time of a few minutes and reduced patient pain [14]. Most PTT strategies depend on constructing nanomaterials utilizing enhanced permeability and retention effects and conjugation with cell-specific target ligands [15,16]. However, the current targeting ability of the injected reagent to reach the lesion site is relatively low [17]. In addition, most PS for PTT has a relatively low photothermal conversion efficiency and required high power of light irradiation for therapy [18].

Recent emerging nanotechnology enables advanced PSs for PDT and PTT to enhance therapeutic performance with reduced adverse effects. However, most PDT and PTT with the state-of-the-art PSs have been applied to oncology to eliminate tumor cells [7,19,20]. On the other hand, phototherapy for non-oncologic applications has less appeared in both preclinical and clinical fields. Relatively small numbers of traditional PSs have been applied to phototherapy as a treatment option for non-cancerous lesions. In this Review, we focus on the non-oncological applications of nanomedicine-based phototherapy. We provide the mechanisms of PDT and PTT, recent PSs with nanocarriers in phototherapy, and a comprehensive overview of clinical trials and preclinical studies using advanced nanomaterials in various non-oncologic diseases.

## 2. Mechanisms of Nanomedicine-Based Phototherapy

Phototherapy-based nanomedicine can be broadly classified into PDT and PTT. In the section, we present the mechanism of phototherapy how to cause selective damage to the target cells with PSs. In addition, we describe nanocarriers which can be conjugated with PSs to enhance phototherapy performance.

### 2.1. Mechanism of Photodynamic Therapy (PDT)

PDT is phototherapy to kill target cells involving light and photosensitizing chemical agents. PDT involves three principle components: (1) PS, a substance, which induces a chemical alteration in the photochemical process, (2) light, and (3) oxygen [21,22,23]. A PS, administered intravenously or topically, accumulates in the target tissue and remains inactive until exposed to the photosensitizing light [8,22,24,25].

**Figure 1:**
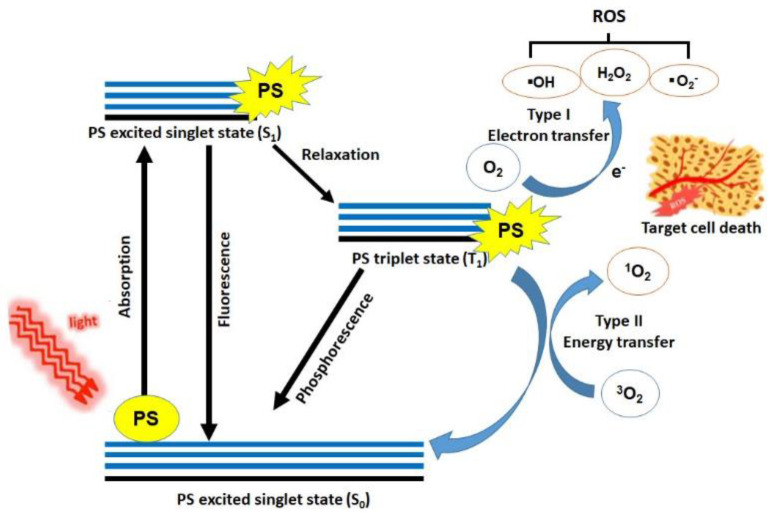
Mechanism of action on target lesion in photodynamic therapy (PDT). There are two types of reaction during PDT. Following absorption of light, a photosensitizer is transformed from the ground state into an excited state. The activated sensitizer generates radicals (type-I reaction) or oxidative substrates (type-II reaction) to damage the cell. Modified from Refs. [29,30,31].

Following the absorption of light, the PS is transformed from its ground state (singlet state, S_0_) into a relatively long-lived electronically excited state (triplet state, T_1_) via a short-lived excited singlet state (S_1_). The lifetime of the triplet state enables the interaction of the excited PS with the surrounding molecules. The activated PS can undergo two types of reactions (Figure 1).

The type I mechanism involves the PSs reacting with biomolecules transferring an electron to form a radical cation. These radicals react with oxygen resulting in reactive oxygen species (ROS) [17,18,19,20]. The Type II mechanism is related to direct energy transfer between the sensitizer′s excited triplet state and the ground state generating singlet oxygen molecules [17,21]. Generally, the Type II process predominates during PDT such that singlet oxygen is the primary cytotoxic agent responsible for biological effects. Also, the Type I reaction is more important at low oxygen concentrations or in more polar environments [22,23].

PDT kills cells by necrosis or apoptosis. Necrotic cells swell and disrupt the plasma membrane, resulting in the release of intracellular components that lead to the inflammatory reaction. Apoptotic cells bleb and shrink with nuclear fragmentation via endonuclease that degrades DNA into oligonucleosomal fragments [26]. PDT can affect discoloration, skin infections, erythema, itching, or burning at target tissue and neighboring normal tissue [27,28].

#### 2.1.1. Light Sources for Photodynamic Therapy

The categories of light sources for PDT include halogen lamps, light-emitting diodes (LEDs), and lasers. The selection of light sources depends on the location of the target and the type of PSs. LEDs have been widely applied with low cost and diverse illumination spectra options due to broad illumination areas, especially for dermatological diseases with 5-aminolevulinic acid (5-ALA) sensitizers in clinics. However, LEDs have poor coupling efficiency for optical fiber delivery or beam collimation to access distant lesions such as the gastrointestinal tract with endoscopy. However, lasers can be used for both superficial and deep tissue PDT applications. Lasers provide high optical power and monochromatic light delivered to a distal irradiation location via optical fiber coupling.

The criteria for selecting an optimal light source are as follows: (1) absorption spectra of reactive PSs, (2) the state of the disease (location and characteristics of the tissue), and (3) cost. Proper dosimetry can affect the treatment efficiency of PDT [8,32]. Dosimetry is classified by the method and requires (1) the total dose of the light source to be irradiated, (2) the irradiation time of the light source, and (3) transmission of the light source [33,34,35]. To improve the efficiency of PDT, it is necessary to accurately predict and verify all therapeutic doses. The influence of the light source on the therapeutic dose is determined by the wavelength and light power. The procedure is performed by calculating the final amount of light according to the lesion’s condition to be treated.

#### 2.1.2. The Role of Oxygen in Photodynamic Therapy

Singlet oxygen, the major reactive oxygen species, causes physicochemical damage to intracellular organelles, such as mitochondria and membrane systems. This leads to target cell death from PDT. Also, only cells proximal to ROS production are directly affected by PDT due to high reactivity and short half-life of ROS. As the half-life of singlet oxygen in biological tissue is <0.04 μs with a limited radius effect (0.20 μm), ROS is limited to the lesion tissue area, where the PS accumulates [22,36]. However, the necrosis of target tissue by singlet oxygen has less effect in the 1-2-log reduction compared to the 6-8-log reduction required for treatment due to uneven distribution of PSs and oxygen in the tissue, and the short “free diffusion length” and photobleaching of ROS [37].

#### 2.1.3. Photosensitizers Used in Photodynamic Therapy

PS is a light-sensitive material that is an essential factor for PDT. PSs can be used because of their toxicity at a specific wavelength [38]. In PDT, PS is activated by absorbing a light source’s specific wavelength irradiated for the destruction of target cells. It plays the role of producing ROS to destroy target cells. PSs rarely exhibit cytotoxicity, even at high concentrations unless exposed to light. However, PSs generate ROS and show toxicity only when excited by light of a specific intrinsic wavelength. Additionally, the triggered ROS induces cell necrosis or apoptosis, damages the extracellular matrix (ECM), and allows for deeper penetration. PS can also be used as a tissue penetration enhancer [39].

Currently, in clinical trials, PS can be divided into porphyrin-based and non-porphyrin-based types. Thus far, the most extensively studied PSs are porphyrins, which were identified in the mid-nineteenth century. 5-Aminolevulinic acid (5-ALA) is a prodrug that converts into protoporphyrin IX in tumor tissues. 5-ALA has been used extensively for PDT in Europe, where it is readily available and inexpensive [24]. To perform PDT with high therapeutic efficiency, the characteristics of PSs to consider are (1) little toxicity when not exposed to light, (2) proper wavelength of NIR light to maximize tissue penetration of light, (3) high singlet oxygen yield, (4) selective accumulation in the lesion [40].

### 2.2. Mechanism of Photothermal Therapy (PTT)

PTT is a treatment that selectively eliminates lesions through a photothermal transducer which converts light into heat [14,41,42].

#### 2.2.1. Phototherapeutic Mechanism in Photothermal Therapy

**Figure 2:**
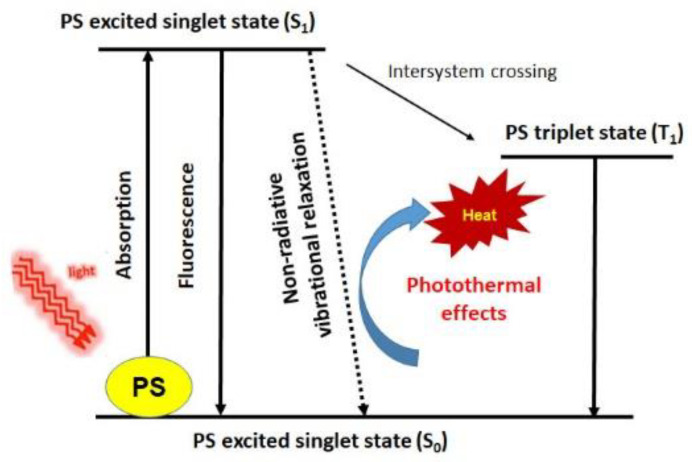
Mechanism of action on non-oncologic diseases in PTT. A lesion containing PTT agents is irradiated with a light source. The radiation absorbed by photothermal agents is converted to heat energy, causing cell death. Modified from Ref. [54].

PTT agents are active substances that generate heat in response to light exposure. When the PTT agents absorb light, electrons transition occurs from the ground state to the excited state (Figure 2). The electronic excitation energy subsequently relaxes through nonradiative decay channels, leading to the overheating of the local environment around light-absorbing materials [43]. This treatment is a highly effective and non-invasive, capable of eliminating target lesions [44]. The generated heat may cause hazardous cellular effects such as protein aggregation and denaturation, cytosol evaporation, and cell lysis for living cells [45]. However, improper irradiation dosing may lead to side-effects, including tissue burning, swelling, and inflammation [46].

Gold nanoparticles have attracted significant interest among PTT agents for nearly 20 years, owing to their unique physicochemical properties such as bacteriostatic, anticorrosive, and antioxidative characteristics [47]. Research on PTT began in the early 2000s; it is now approved by the US FDA. Preclinical and clinical studies are being conducted on products such as AuroLase [48]. The agents can be natural chromophores in the tissue or externally added dye molecules such as indocyanine green (ICG) and porphyrins coordinated with transition metals [49,50]. However, natural chromophores suffer from very low light absorption.

#### 2.2.2. Photosensitizers Used in Photothermal Therapy

The selection of photothermal agents is based on their strong absorption cross-sections and highly efficient light-to-heat conversion. Recently, several nanoparticles were used for photothermal treatment. Metal nanoparticles show four to five times more light absorption than light-absorbing dyes. This strong absorption can be effectively treated using a laser with less energy. Therefore, it is possible to reduce the destruction of normal surrounding tissues. Besides, metal nanoparticles have high stability against light, and there is no loss of fluorescence. The gold nanoparticles in the body adhere to the target cells. When the laser is irradiated into cells, heat is generated killing the target cells [51,52]. It can absorb most of the energy in the visible and NIR regions by controlling the gold nanoparticle’s size, shape, and maternity.

The gold nanospheres react to light in the visible region to create strong surface plasmon resonance (SPR). As the particle size increases, the wavelength of the reacting light moves to the longer side and reacts to light in the NIR region [14,53]. In addition, when gold nanoparticles change from a spherical to rod shape, SPR formed in the nanoparticle’s longitudinal direction reacts to light in the NIR region. The reaction frequency of nanoparticles can be controlled by changing their size and shape. Studies on gold nanoparticles that can use NIR rays with high permeability in tissues are being conducted.

### 2.3. Nanocarriers for Phototherapy

A nanocarrier is a nanomaterial with 1–100 nm to deliver substances such as photosensitizers. The intrinsic characteristics of PSs with low water solubility and aggregating tendency lowers the therapeutic efficacy of phototherapy [55]. In order to improve the delivery of PSs, various kinds of nanocarriers have been developed. In this section, we introduce the latest nanocarriers in phototherapy.

The colloidal carriers are frequently used in the drug delivery system by protecting the drug against degradation while preventing the drug’s adverse side effects and toxicity [56]. The polymer-drug conjugates, polymeric micelles, and liposomes are examples of colloidal carriers. The polymeric micelles consist of a hydrophilic stealth corona and a hydrophobic core suitable for accommodating hydrophobic drugs [57]. It can protect the drug from harsh biological environments, such as low pH and hydrolytic enzymes, significantly improving the water solubility of the hydrophobic drug and facilitating drug targeting by its small size [58]. Liu et al. studied PDT with polymeric micelles with the loading of 5,10,15,20-tetrakis(m-hydroxyphenyl)chlorin (mTHPC) [57]. The epidermal growth factor receptor (EGFR)-targeted nanobody was added to the micelles to enhance targeting function. In addition, the mTHPC-loaded micelle showed prolonged blood circulation time than free mTHPC. Other photosensitizers, such as ICG and phthalocyanine, were encapsulated into micelles and applied to PDT/PTT [59,60,61]. Therefore, polymeric micelles are promising nanocarriers of PS in phototherapy.

Liposomes are spherical-shaped, small artificial vesicles created from phospholipids and cholesterol [62]. They have higher biocompatibility and biodegradability, trapping the hydrophilic and lipophilic drugs with low toxicity [63]. Therefore, PS-encapsulated liposomes were used in phototherapy [64,65]. A single PS or dual PSs, or other chemotherapeutic drugs have been encapsulated within liposomes [66,67].

Polymeric nanocapsule (NC) consists of a liquid/solid core coated with a polymeric shell [68]. NC can effectively increase drug-loading efficiency with reduced polymeric matric contents of the nanoparticles compared with polymeric nanospheres [69,70]. The poly(lactic-*co*-glycolic acid) (PLGA) is the US Food and Drug Administration (FDA)-approved polymers for human use [71]. PLGA has excellent biocompatibility and tunable biodegradability. Therefore, PLGA-encapsulating PS has been used for phototherapy recently [72,73]. Moreover, the polymeric NC has shown enhancement of therapeutic efficacy by the co-encapsulating PS and chemotherapeutic agents [74].

Carbon-based materials are popular in chemistry and biomaterials due to their environmental friendliness [75]. They possess high mechanical strength, good biocompatibility, tunable cavity, controlled release manner, and low toxicity suitable for biomedical applications [76]. Besides, carbon-based material has photodynamic or photothermal properties due to its distinctive structures [77]. Therefore, various carbon-based nanocarriers including carbon nanosheet, carbon dot, graphene, carbon nanotube, and fullerenes, have shown significant theranostic performances in phototherapy [78,79,80,81].

Metal-based nanomaterials have drawn great attention with their tunability in electronic and electro-optical properties and high luminescence [82]. The gold nanomaterials can be synthesized with different forms and dimensions and easily functionalized by all kinds of biomolecules with biocompatibility [83]. Therefore, gold-based nanomaterials were conjugated with PSs or drugs for biomedical application [84,85,86]. On the other hand, magnetic nanoparticles have been employed as carriers for drugs. Magnetic nanoparticles such as magnetite (Fe_3_O_4_) can be delivered to the desired region under an external magnetic field [87]. PS-conjugated magnetic nanoparticles have been used for PDT [88]. In particular, magnetic iron oxide nanoparticles (MIONs) possess several important properties, including small size, biocompatibility, chemical compatibility for biomedical applications. Due to the proton’s short transverse relaxation time (T2), MIONs can be used as an MRI contrast agent [89]. A recent study with ICG-conjugated MIONs reported PTT efficacy with multiple imaging modalities, including MR, ultrasound (US), and fluorescence [90].

The upconversion nanoparticles (UCNP) are another promising nanocarrier in the phototherapy field. Especially, the rare-earth-doped near-infrared (NIR)-to-visible UCNP has a promising potential [91]. Due to the two-photon or multiphoton mechanisms, UCNP can convert long-wavelength radiation into shorter-wavelength emission [92]. The NIR light irradiation enhanced tissue penetration than visible light with reduced phototoxicity and background autofluorescence [93]. Therefore, UCNP-based PS was applied to the phototherapy to increase therapeutic efficacy [94]. Another recent study used the conjugation of UCNP with dual PSs (chlorin e6 and Rose Bengal) for greater ROS generation than single PS-based PDT [95].

With nanotechnology, conjugated PDT or PTT agents showed enhanced therapeutic efficacy with improved drug delivery to the target tissue. Also, nanocarrier-conjugated PSs can be visualized with multiple imaging modalities, including fluorescence. The recent representative examples of PS-conjugated nanocarriers for phototherapy is shown in Table 1.

## 3. Non-Oncologic Applications of Photodynamic Therapy

**Figure 3:**
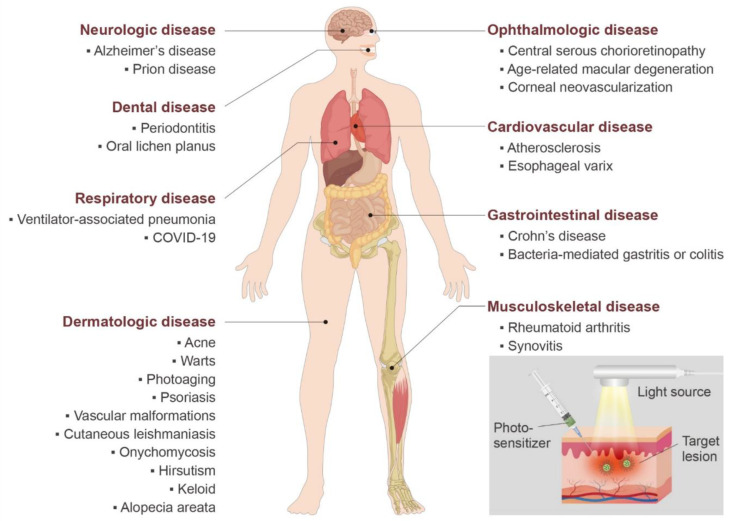
Non-oncological application of PDT. Various human diseases other than cancer that can be treated with PDTs are categorized based on different organ systems. The gray box describes the process of PDT including a light source with an appropriate wavelength, photosensitizers which can be applied topically (as in the figure) or accumulated to the target region via intravenous injection, and the irradiated target region where the phototoxicity is produced by the photoactivation of photosensitizers.

While PDT has been used in many cancer treatments, here we describe PDT’s non-oncologic applications focusing on clinical study. Some preclinical studies which can be translatable to clinical practice are also included. We illustrate various non-cancerous human diseases that can be treated with PDT in Figure 3.

### 3.1. Dermatologic Disease

#### 3.1.1. Acne

Acne is a disease of pilosebaceous units [96]. This is a major dermatologic disorder that occurs in adolescents and young adults. The factors affecting the pathophysiology of acne vulgaris include follicular hyperkeratosis and occlusion [97], decreased linoleic acid [98], androgen stimulation, bacterial, hereditary, and immunological factors [99]. PDT with topical porphyrin precursors showed a good therapeutic response to acne. It promotes antimicrobial and anti-inflammatory effects, inhibition and destruction of sebaceous glands, and enhanced epidermal turnover promoting reduced follicular obstruction [100].

Yang et al. performed a prospective clinical trial with 75 patients with acne conglobata, a severe form of cystic acne that is difficult to manage [101]. The 5-ALA PDT therapy group showed significant improvement in acne lesions and reduced scar formation compared to the control group. Although extensive studies support the PDT’s effect on acne, consensus on the optimal therapeutic protocol is necessary [102].

#### 3.1.2. Warts

Conventional therapeutic methods (e.g., glutaraldehyde and cryotherapy) for hand and foot warts may cure up to 70% of warts in 3 months [103]. However, some recalcitrant warts remain despite treatment. Stender et al. performed a randomized double-blind trial with 5-ALA [104]. The PDT group showed a significant reduction in the wart area than the placebo PDT group at 14 and 18 weeks after treatment.

Genital warts are related to human papillomavirus (HPV) infection and sexually transmitted diseases. Liang et al. performed a randomized clinical trial in patients with condylomata acuminata (CA) [105]. The 5-ALA PDT group showed a lower recurrence rate than the CO_2_ laser therapy group (9.38% vs. 17.39%, *p* < 0.05). However, owing to the absence of the optimized therapeutic protocol, PDT is less frequently used in clinics, despite the positive results.

#### 3.1.3. Photoaging

Aging affects all skin constituents, resulting in reduced generation of the dermal matrix [106]. Nonablative treatment of photoaging has become more popular, owing to its reduced side effects. PDT with a topical PS is applied for the same purpose [107]. Shin et al. performed a randomized controlled split-face study on Asian skin [108]. They used 5-ALA liposomal spray to treat periorbital wrinkles. The 5-ALA PDT group showed better results in wrinkle reduction than long-pulsed Nd:YAG laser therapy. However, further clinical trials with optimizing parameters and protocols are needed.

#### 3.1.4. Psoriasis

Psoriasis is a chronic inflammatory skin disease mediated by increased keratinocyte proliferation and T-cell infiltration [109]. PDT with 5-ALA, methylene blue, verteporfin, and hypericin were applied to treat psoriasis [110]. However, the clinical trial did not show a significant treatment benefit compared to the control group [111]. Therefore, the guidelines of care for the management and treatment of psoriasis with phototherapy from the American Academy of Dermatology and the National Psoriasis Foundation do not recommend topical 5-ALA PDT or methyl-aminolevulinate (MAL) PDT for localized psoriasis [112].

#### 3.1.5. Vascular Malformations

Vascular malformations are abnormalities of vasculatures, including venous, arteriovenous, capillary, and lymphatics. Port-wine stain (PWS) is a congenital vasculopathy owing to an abnormal capillary network in the upper dermis with a normal overlying epidermis [113]. It becomes darker and thicker with age. Zhao et al. performed a randomized controlled trial for PWS patients with hemoporfin [114]. The hemoporfin (5 mg/kg) was transfused to the patients at a constant rate over 20 min. The target site was irradiated with a 532 nm continuous laser for 20 min with a power density of 80–100 mW/cm^2^. The therapeutic efficacy was evaluated eight weeks after PDT. The PDT group showed a higher improvement rate than the placebo group (89.7% vs. 24.5%, *p* < 0.0001).

Jerjes et al. performed a clinical trial for patients with vascular tumors (hemangioma) or vascular malformation [115]. The mTHPC was used as a PS. mTHPC (0.15 mg/kg) was administered intravenously to the patients 96 h before treatment. The light was delivered by a needle-type optical fiber into the interstitium under ultrasound guidance to treat deep-seated malformation. Among the treated patients, 22 of 43 showed a good therapeutic response by clinical assessment after PDT. Although PDT was not superior to other treatment modalities to manage vascular malformations, additional advantages of the PDT were demonstrated including less invasiveness, repeatability, and low residual toxicity.

#### 3.1.6. Cutaneous Leishmaniasis

Cutaneous leishmaniasis (CL) is a parasitic disease of the skin. It is caused by female sandflies infected by Leishmania species [116]. The goal of treating CL is the eradication of amastigotes and reduction of lesion size with minimal scarring [117]. Although the paromycin ointment was suggested as the first-line treatment, the optimal therapeutic regimen has not been established. Therefore, several studies reported the use of PDT as a treatment option for CL [118]. A placebo-controlled, randomized clinical trial was undergone to treat CL [119]. In the PDT group, the lesion was irradiated 4 h after the application of the 5-ALA cream. The PDT group showed a higher rate of improvement over a paromycin ointment group and the placebo group (93.5% vs. 41.2% and 13.3%, respectively, *p* < 0.001). Also, all lesions that underwent PDT showed a parasitological cure. Therefore, PDT with topical PS could be an alternative therapeutic modality in CL patients.

#### 3.1.7. Onychomycosis

Onychomycosis is a fungal infection that causes discoloration, thickening, and separation from the nail bed [120]. PDT has become popular based on successful in vitro studies [121]. Sotiriou et al. underwent a single-center clinical trial in 30 toenail onychomycosis patients [122]. 5-ALA was topically applied to the nail bed and PDT was done 3 times every other week. After one year of PDT therapy, 13 of 30 (43.3%) patients showed cure and the cure rate fell to 36% at 18 months. Gilaberte et al. performed a randomized controlled clinical trial with a PS, methy aminolevulinate (MAL) [123]. The MAL-PDT group did not show significant differences with the placebo PDT group. In the ancillary analysis, onychomycosis without dystrophy showed a better clinical response and microbial cure rate than the dystrophy group in MAL-PDT. Therefore, the use of PDT for onychomycosis is recommended in cases where conventional therapy fails or when patients cannot endure adverse effects of standard drug [102].

#### 3.1.8. Hirsutism

Hirsutism is excessive hair growth in women in places usually associated with androgen-dependent areas of the body, including the face, chest, abdomen, lower back, upper arms, and thighs [124]. Generally, it is managed by the mechanical removal of excess hairs, suppressing ovarian androgen production, and anti-androgen medication. ALA-based PDT showed therapeutic efficacy in patients with hirsutism [125]. Comacci et al. applied PDT to patients with hirsutism to remove excess hairs [126]. After 5-ALA was topically applied to the lesion, the patients showed a 75% hair reduction in 12 months after PDT. PDT was found more effective for actively growing phased (anagen) hairs. The cytotoxic effect in hair bulge and papilla with local inflammation was proposed as a potential mechanism of the PDT-induced epilation [126].

#### 3.1.9. Keloid

The development of keloids and hypertrophic scars is related to impaired fibroblastic proliferation and collagen deposition after trauma, inflammation, surgery, or burns [127,128]. They can occur in genetically susceptible individuals [129]. Keloid removal by surgical excision alone leads high recurrence rates of 45–100% [130]. Two clinical PDT case reports used MAL-based PDT as an alternative treatment [131,132]. After PDT, the keloid area was significantly softened and reduced in volume. Although the actual mechanism was not well known, the PDT cytotoxicity damaged target tissue resulting in necrosis and apoptosis, microcirculation arrest, immune response induction, and inflammation [132]. With further study by optimizing therapeutic protocols, PDT could be a potentially effective keloid therapy.

#### 3.1.10. Alopecia Areata

Alopecia areata (AA) is a complex genetic, immune-mediated disease that affects hair follicles and results in nonscarring hair loss [133]. Several clinical studies have attempted PDT to treat AA and made controversial results. Linares-González et al. performed 5-ALA-based PDT on the refractory form of AA patients [134]. After the monthly session of PDT for 6 months, a regrowth of scalp hair was observed and there were no relapse 4 months after the end of treatment. However, another previous case report failed to show significant improvement in the PDT group in AA patients [135]. Giorgio et al. showed an additional therapeutic benefit of PDT when combined with the roller therapy [136]. Therefore, PDT may provide benefit to AA which does not improve with conventional treatment.

### 3.2. Ophthalmologic Disease

#### 3.2.1. Central Serous Chorioretinopathy

Central serous chorioretinopathy (CSC) is characterized by a localized, serous detachment of the neurosensory retina in the macular region, and occasionally associated with detachment of the retinal pigment epithelium [137]. Although the exact mechanism of PDT on CSC is not well-known, PDT may decrease choroidal hyperpermeability by inducing choriocapillaris damage and vascular remodeling [138]. Van Dijk et al. performed a multicenter randomized controlled trial with verteporfin [139]. The PDT-treated group showed a significantly higher proportion of complete subretinal fluid resolution than the high-density subthreshold micropulse laser treatment group (67.2% vs. 28.8%, *p* < 0.001). In addition, visual acuity and retinal sensitivity were improved in the PDT-treated group.

#### 3.2.2. Age-Related Macular Degeneration

Age-related macular degeneration (AMD) is a chronic structural change in the macular area under multifactorial interaction of metabolism, functions, genetics, and the environment [140]. The most common cause of vision loss is the development of choroidal neovascularization, which is a common type of AMD [141]. In randomized controlled clinical trials, PDT is less effective than antivascular endothelial growth factor (Anti-VEGF) agents [142]. Another clinical trial showed that the combination therapy group of an anti-VEGF agent and PDT showed a better therapeutic effect than the monotherapy groups [143]. Therefore, combination therapy with PDT and anti-VEGF agents should be considered for treating eyes with choroidal vasculopathy.

#### 3.2.3. Corneal Neovascularization

Corneal neovascularization is characterized by abnormal proliferation of preexisting blood vessels and lymphatic vessels into the corneal stroma [144]. Increased vascular permeability leads to corneal scarring, edema, lipid deposition, and inflammation, resulting in permanent visual loss [145]. The regression of corneal neovascularization with PDT was quantitatively analyzed in a clinical trial [146]. After one month of PDT with verteporfin, eight were occluded among 25 new vessels, and 15 were partially occluded (regression ranges 15.3% to 85.1%), and two vessels showed worsening. The mean areas of corneal neovascularization were decreased by 70% after PDT. Moreover, a randomized controlled trial revealed that combination therapy with verteporfin PDT and an anti-VEGF agent showed a significant reduction in corneal neovascularization area [147]. However, the number of enrolled patients was small (7 patients); thus, further clinical trials with larger sample size will be needed to confirm the results.

### 3.3. Cardiovascular Disease

#### 3.3.1. Atherosclerosis

The application of PDT for atherosclerotic plaque treatment has limitations, including (1) nonspecific accumulation of the PS in the skin, leading to cutaneous photosensitivity [148]; (2) relatively long drug-light interval (from 3 to 24 h) after systemic injection with most of the tested PSs [149]; and (3) difficulty of light delivery into the targeted vessel. However, 5-ALA PDT was used as an adjuvant therapeutic modality of angioplasty to prevent restenosis in a clinical trial [150]. PS motexafin lutetium was used in another phase I clinical trial on patients with peripheral arterial atherosclerosis [151]. The patients received motexafin lutetium one day before photoangioplasty. A laser-delivering fiberoptic catheter was positioned to stenosis lesion under fluoroscopic guidance during angioplasty. There was no evidence of significant, dose-limiting systemic toxicity. Other types of PSs, including photofrin, phthalocyanine, verteporfin, and ICG, were also evaluated to treat atheromatous plaques indicating potential prevention of neointimal hyperplasia [152].

#### 3.3.2. Esophageal Varix

The esophageal varix is the dilated veins that bulge into the lumen, producing an uneven wormlike surface inside the esophagus [153]. PDT can selectively damage the vascular endothelial cells and result in blood flow stasis, followed by thrombosis, vascular occlusion, and eventually, the destruction of the abnormal microvasculature [154]. Li et al. performed a randomized controlled trial in 14 patients [155]. After 3 months of hematoporphyrin monomethyl ether (HMME) PDT, the number of newly visible vessels was significantly decreased in the PDT-treated group than in the control group. The recurrent bleeding rate was significantly lower in the PDT-treated group than in the control group. Therefore, it can be a potential therapeutic modality to treat newly visible vessels and prevent recurrent bleeding from esophageal varix.

### 3.4. Dental Disease

#### 3.4.1. Periodontitis

Periodontitis is an inflammatory disease caused by dysbiotic dental biofilm and characterized by progressive destruction of the periodontium [156]. PDT was applied as an antimicrobial therapy to treat biofilm-mediated diseases [157] as the PDT-induced free radicals and singlet oxygen are toxic to bacteria [158]. However, a recent meta-analysis with a randomized controlled study showed a lack of clinical benefit compared to conventional treatments in periodontitis [159]. Another recent clinical trial applied ICG-PDT as an adjunct modality to periodontitis patients with scaling and root planning (SRP), a current standard treatment [160]. The ICG-PDT group showed a significant improvement in periodontal probing depth and clinical attachment level compared to the SRP group.

#### 3.4.2. Oral Lichen Planus

Oral lichen planus (OLP) is a common T-cell-mediated inflammatory disorder that affects the oral mucosa [161]. Although corticosteroids, immunosuppressants, or immunomodulatory agents are used to treat OLP, PDT can be used as an alternative treatment modality. Aghahosseini et al. underwent PDT in OLP patients with methylene blue as a PS [162]. Four out of five OLP lesions displayed clinical improvement after PDT. A recent prospective, case-controlled study performed PDT with phenothiazine chloride as a PS [163]. The PDT was performed in 4 sessions on days 1, 3, 7, and 14 resulting in a significant reduction of lesion size, improvement of Autoimmune Bullous Skin Disorder Intensity Score (ABSIS) and Thongprasom-scores. The quality-of-life parameters also showed significant improvement in the PDT group. Therefore, PDT can be a therapeutic option in OLP patients.

### 3.5. Neurologic Disease

#### 3.5.1. Alzheimer’s Disease

Alzheimer’s disease (AD) is a common progressive neurogenerative disorder with abnormal accumulation of beta-amyloid (Aβ) plaque as a characteristic finding [164]. The inhibition of Aβ aggregation is a potential treatment intervention for AD inhibition of the Aβ aggregation is a potential treatment intervention for AD [165]. Several studies have attempted to disaggregate Aβ in preclinical situations with Rose Bengal [166], methylene blue [167], or 5,10,15,20-tetrakis(4-sulfonatophenyl)porphyrin (TPPS) [168]. These preclinical studies lead to the inhibition of Aβ aggregation in vitro and in vivo in a Drosophila AD model. Further studies are needed to boost the PSs in the AD model.

#### 3.5.2. Prion Disease

Prion disease is a fatal neurodegenerative disease including Creutzfeldt-Jacob disease (CJD) and kuru in humans, scrapie in sheep, and bovine spongiform encephalopathy (BSE) in cattle [169]. They are transmissible within and between mammalian species and caused by the conversion of a natively occurring prion protein (PrP^C^) into its misfolded infectious form (PrP^TSE^) [170]. The prevention of the action of neurotoxic species of prion disease is the therapeutic goal. Kostelanska et al. used PDT to treat prion disease with a phthalocyanine PS in a preclinical study [171]. PDT inhibited the infectious form of the prion protein in mouse brain homogenate. Therefore, PDT suggests a promising approach to inactivate the misfolded infectious form of the prion protein.

### 3.6. Skeletal Disease

#### 3.6.1. Rheumatoid Arthritis

Rheumatoid arthritis (RA) is a systemic, inflammatory autoimmune disease currently considered a disease of the joints [172]. Synovectomy is an invasive and destructive procedure that requires long periods of rehabilitation. A preclinical PDT study showed cell death in cells involved in inflammation and hyperplasia in the joint [173]. Interestingly, Hendrich et al. examined the feasibility of conventional drug to treat RA as a PS [174]. The in vitro cytotoxicity of laser-irradiated chloroquine or methotrexate was more than 20 times compared to drug-treated or laser irradiation alone in human synovial fibroblasts from RA patients. The high grade of vascularization involved in RA would enable the accumulation of a PS into the inflamed tissue. Further clinical trials would be necessary in the future.

#### 3.6.2. Synovitis

Chronic synovitis is a pathologic feature of RA, osteoarthritis, spondylarthritis, and villonodular synovitis [175]. The residual tissue after synovectomy may lead to recurrent synovitis. Dietze et al. underwent in vitro and rat in vivo studies that showed significant 5-ALA accumulation in the inflamed synovial tissues [176]. Kirdaite et al. showed a higher accumulation of 5-aminolevulinic acid hexyl ester (h-ALA) from RA patients’ tissue [175]. The microscopic image showed the localized accumulation of the protoporphyrin IX in the synovial lining layer, endothelial cells, and macrophages. In addition, the PDT-induced cytotoxic effect was observed via Sytox green staining. Therefore, PDT may be used as a less invasive treatment method for synovitis with a high degree of specificity. Further human clinical trials are warranted to find a therapeutic efficacy.

### 3.7. Gastrointestinal Disease

#### 3.7.1. Crohn’s Disease

Crohn’s disease is a relapsing inflammatory disorder that potentially affects the entire gastrointestinal tract and presents with abdominal pain, fever, bowel obstruction, or bloody or mucus diarrhea [177]. Fabre et al. used low-dose delta-ALA-PDT in a mouse colitis model. [178]. The PDT group showed improvement in the colitis score, decreased proinflammatory cytokines, interleukin-6, 17, and interferon-gamma. Therefore, PDT has therapeutic potential against inflammatory bowel disease by modulating the local immune system.

#### 3.7.2. Bacteria-Mediated Gastritis or Colitis

*Helicobacter pylori* is a human pathogen that colonizes the gastric mucosa and causes chronic infection. Baccani et al. used porphyrin-PDT as an adjuvant with conventional doxycycline therapy [179]. The results showed that combination therapy with PDT and doxycycline showed a higher antibacterial effect than monotherapy.

Cassidy et al. performed targeted PDT to treat colon-residing bacteria [180]. The h-ALA, methylene blue, and 5,10,15,20-tetrakis(1-methyl 4-pyridinio)porphyrin tetra(p-toluenesulfonate) (TMPy) were tested for colon-targeted delivery. Among the results, PDT with h-ALA and oxygen releasing compound reduced up to 7.73 logs of *Bacteroides fragilis*, which causes chronic infection of the colon. Therefore, PDT may be used with targeted PSs in the gastrointestinal tract to kill specific pathogens.

### 3.8. Respiratory Disease

#### 3.8.1. Ventilator-Associated Pneumonia

Ventilator-associated pneumonia (VAP) is a life-threatening infectious disease related to patients who require mechanical ventilation [181]. The endotracheal tube (ET) is the major cause of VAP. Methylene blue-PDT reduced the ET tube polymicrobial biofilm by more than 99.9% after a single treatment [182]. Recent work showed that curcumin-containing ET reduces gram-negative and gram-positive bacteria by up to 95% [183]. Therefore, PDT has the potential as a preventive modality against VAP.

#### 3.8.2. COVID-19

COVID-19 is caused by an infection related to the severe acute respiratory syndrome coronavirus 2 (SARS-CoV-2) virus strain. Although there are few relevant in vitro or in vivo studies of COVID-19, PDT can be a potential therapeutic strategy [184]. Moghissi et al. attempted methylene blue-PDT for COVID-19 patients [185]. Dias et al. suggested that PDT could decrease the microbial load in the respiratory tract using the nebulization of PSs [186]. Recent reports showed that methylene blue has an inhibitory function of the SARS-CoV-2 virus in vitro at a lower concentration than hydroxychloroquine or azithromycin [187]. Further clinical studies are needed to evaluate the therapeutic efficacy of PDT on COVID-19.

Overall, we reviewed the various kinds of non-oncologic PDT applications in clinical and potentially promising preclinical studies summarized in Table 2. The 5-ALA or MAL was used to treat dermatologic applications, including acne, warts, photoaging, cutaneous leishmaniasis, and onychomycosis. The representative clinical trials showed significant clinical improvements when compared with control or placebo groups. In ophthalmologic applications, verteporfin was applied as PSs with laser for localized illumination. The results showed a better clinical response of PDT treated group than high-density subthreshold micropulse laser therapy in central serous chorioretinopathy. However, PDT showed additional therapeutic benefit only when combined with anti-VEGF therapy in age-related macular degeneration and corneal neovascularization. In esophageal varix, the HMME-based PDT group showed a reduction of neovascularization. ICG was applied to treat periodontitis and showed additional therapeutic effects when combined with standard SRP treatment. In sum, PDT brought clinical benefit as an individual or adjuvant therapeutic modality to treat non-oncologic diseases in clinical trials. However, the number of enrolled patients was limited. Therefore, extended clinical trials with a large population will warrant clinical efficacy of PDT.

## 4. Non-Oncologic Applications of Photothermal Therapy

### 4.1. Atherosclerosis

Reducing the burden of atherosclerosis below the Glagov threshold is a therapeutic target for cardiovascular disease [188]. Kharlamov et al. used silica-gold nanoparticles to treat coronary artery stenosis [189]. Plasmonic photothermal therapy was applied to patients with coronary artery disease. Nanoparticles were delivered to the atheroma via a bioengineered patch, and the lesion was irradiated by an intravascular NIR laser. After 12 months, the mean reduction of total atheroma volume was significantly reduced over the control group. The event-free survival was significantly lower than that of the other groups without any target lesion-related complications. Therefore, silica-gold nanoparticle-based photothermal therapy can be employed for patients with coronary artery disease to treat atherosclerosis.

### 4.2. Dry Eye Syndrome

The dry eye occurrence has increased due to the substantial screen time watching a computer monitor, tablet, or smartphone. To treat dry eye syndrome, Pang et al. developed a gold nanoparticle-based hydrogel patch that can attach to the skin of the lacrimal gland [190]. After watching videos for 3 h, the patch-attached eye showed increased eye-protective results over the control eye. The infrared camera showed an increased temperature of the patch lesion. These types of noninvasive biocompatible patches can be applied to treat dry eye syndrome.

## 5. Future Perspective of Nanomaterials for Non-Oncologic Disease

Current PS agents suffer from low target sensitivity and specificity with off-target toxicity. In addition, clinical PDT/PTT efficacy is restricted by limited tissue penetration of photosensitizing light due to absorption and scattering within the tissue. The application of phototherapy will expand when these limitations are resolved. In this section, we introduce strategies with the state-of-the-art nanomaterials, that have been mostly applied to oncology, to improve phototherapy in non-oncologic diseases as a future direction.

### 5.1. Multifunctional Nanomaterials for Phototherapy

The residual tissue after PTT tend to regrow by acquired thermal resistance. To resolve this, there has been an attempt to enhance the therapeutic effect by combining PTT and PDT [191]. The results of combination therapy showed enhanced therapeutic efficacy with reduced side effects [192]. Further, the photothermal effect can generate acoustic waves that can be detected and converted into imaging signals, such as in photoacoustic imaging (PAI) [14,193].

To increase both diagnostic and therapeutic efficacy, multifunctional theranostic nanomaterial platform has been developed. Cheng et al. developed a core-shell nanohybrid for multimodal image-guided combined PTT/PDT in CT26 tumor-bearing mice [194]. The nanomaterial is a controllable coating of a zirconium-porphyrin (PCN) shell on Prussian blue (PB) nanoparticles, which show enhanced photodynamic therapeutic effects against hypoxic target cells. In addition, nanocomplex can be used for magnetic resonance imaging, PAI, and fluorescence imaging. The designed integration of diketopyrrolopyrrole (DPP) and benzothiadiazole (BT) molecule dye is a NIR-II fluorescence/PA dual imaging agent that serves PTT and PDT. DPP-BT shows strong absorption in the NIR-I region and fluorescence emission in the NIR-II region [195]. The integration of Cu^2+^ into black phosphorus@Cu nanostructures enabled chemodynamic therapy and enhanced PTT to improve photothermal stability with positron emission tomography (PET), allowing in vivo real-time and quantitative tracking for diagnosis [196]. Therefore, the combination of PTT and PDT is desirable strategy for increasing therapeutic outcome.

### 5.2. Photoactivatable Nanomaterials for Phototherapy

Several nanomaterials show poor signal-to-noise ratio (SNR) with side effects, owing to nonspecific biodistribution and “always-on” pharmacological activities [197,198]. Stimulated responsive nanomaterials have been developed to tackle these challenges, such that the nanomaterials can only be “turned on” in specific external responses. Photoactivatable therapeutic agents have been developed to achieve accurate lesion-specific release and activation. They are chemically functionalized to be “inert” and can be converted into an “active” state by internal or external stimulation [199]. Upon integrating photoactivatable nanomaterials, they can be delivered to specific target tissues through passive or active targeting. After localizing the targeted tissue, the nanocarriers undergo structural changes and generate a PDT/PTT effect with irradiation [200]. The nanomaterial is composed of three components: active-inhibited therapeutic molecules, photoconverting agents, and light-related responsive components. Compared to passive delivery, photoactive nanomaterials offer the possibility of tailoring the release kinetics of the encapsulated active molecules, which is of considerable clinical relevance for targeted delivery to specific lesion areas. In addition, photoactivatable chemotherapy can provide spatiotemporal control over drug activation beyond conventional chemotherapy.

There remain various challenges for clinical translation with sufficient therapeutic efficacy. Many photoresponsive linker’s biocompatibility and their degradation byproducts are currently less understood and require further investigation. Besides, as UV and visible light has limited tissue penetration, NIR-I (700–950 nm) light can be used instead. However, NIR-I nanomaterials have relatively low sensitivity and effective therapy requires high photoirradiation power [201]. With further development, photoactivatable nanomaterials can be promising agents for non-oncologic phototherapy.

### 5.3. Target-Specific Nanomaterials for Phototherapy

The induction of immunogenic cell death (ICD) presents a therapeutic modality, which is attributed to immune system’s ability to eradicate target cells through a “bystander effect” [202]. This ICD can be triggered by ROS production and endoplasmic reticulum (ER) stress. However, most radiotherapy, chemotherapy, and non-targeted PDT cannot induce effective ICD, owing to secondary or collateral ER stress effects [203]. Therefore, a direct ER stress inducer is required for effective ICD. Combination of PDT and PTT has been extensively studied to establish effective nanotherapeutics under light irradiation [204]. The drawback of conventional PDT is its oxygen-consuming process [205]. Low oxygen levels severely limit the production of ROS in PDT, thus weakening ROS-based ER stress and ICD effects [206]. To overcome this limitation, recent report introduced the combination of ER targeting PSs and oxygen-delivering nanomaterials [207]. The ER-targeting pardaxin (FAL) peptides were conjugated with indocyanine green and gold nanospheres, together with an oxygen-delivering hemoglobin liposome to increase ER stress. Still, the mechanisms by which the nanomaterials stimulate the immune response remain poorly understood.

Moreover, the conventional PDT family lacks hydrophilicity. Aza-boron-dipyrromethene (aza-BODIPY) molecules were fabricated into hydrophilic nanoassemblies, contributing to enhanced target tissue accumulation with prolonged blood circulation. The aza-BODIPY family comprises organic PSs with NIR optical characteristics [208]. Chen et al. showed hydrophilic nanomedicines that selectively target sites by aza-BODIPY-encapsulated PS. This was enabled by an enhanced permeability and retention effect to improve diagnosis and therapeutic efficacy [209]. Aza-BODIPYs exhibit good biocompatibility and intense red-shifted NIR absorbance.

### 5.4. Deep Tissue Penetrating Nanomaterials for Phototherapy

Conventional PDT and PTT cannot reach deep-seated target lesions due to insufficient light penetration into the tissue [210,211]. Also, the lesion’s unfavorable physiological environment, such as high interstitial fluid pressure and dense extracellular matrix hinders sufficient PS distribution [212,213]. NIR light (700–1700 nm) has much greater body transparency than visible light. Particularly, the NIR-II (1000–1700 nm) wavelength light can offer deeper tissue penetration due to reduced photon scattering and tissue background [214,215]. The PTT agents activated by NIR-II light, such as graphene or carbon-based nanomaterial conjugated polymer particles have been developed [216,217,218]. Another nanomolecule known as a PTT agent with its high photothermal conversion efficiency, copper sulfide (CuS), has been explored for PDT with their strong absorbance in the NIR-II window and low off-target toxicity [219,220]. Recently a BSA-stabilized CuS nanomolecule combined with chemotherapeutic agent (doxorubicin) showed a promising therapeutic effect of PTT/PDT and doxorubicin [221]. Therefore, the use of NIR-II responsive nanomaterial can further enhance the therapeutic performance of the nanomedicine-based phototherapy.

**Figure 4:**
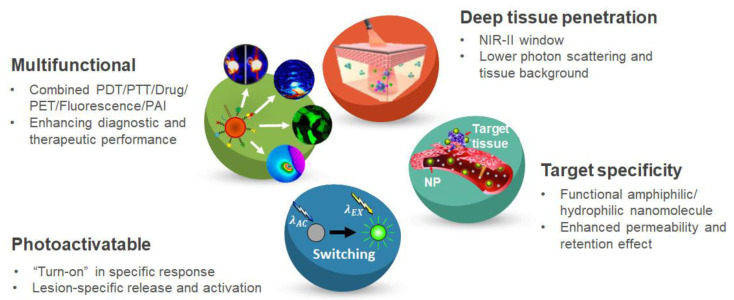
Promising strategies of novel nanomaterials for effective non-oncologic phototherapy.

The strategies used to improve phototherapy such as PDT and PTT with novel nanomaterials are summarized in Figure 4. These provide a rational design of nanomaterials for treating non-oncologic diseases with enhanced theranostic performance of nanomedicine-based phototherapy.

## 6. Remaining Issues

The nanomedicine-based phototherapy for treating various diseases shows promising results in clinical and preclinical studies. While there exist many promising phototherapy results, several limitations still hinder the wide-spread use of phototherapy. (1) The therapeutic phototherapy protocols have not been well established. A standardized protocol is necessary to obtain consistent therapeutic responses; (2) Some clinical studies did not directly compare with the control (or placebo) group. The number of enrolled patients in the randomized controlled trial was too small to prove clinical significance (Table 2). A well-designed, case-controlled clinical trial with a larger population will be needed to confirm the efficacy of phototherapy; (3) The number of clinically applicable PSs is still limited (Table 2). There is a need to develop more PS agents with high target sensitivity and specificity, deeper tissue penetration, and low toxicity; (4) Detailed mechanisms of phototherapy for various non-oncologic diseases are largely unknown due to complex immune reactions in different tissue microenvironment. Further studies with advanced nanomaterials will provide patients with further treatment options for intractable non-oncologic diseases with refined phototherapy.

## Figures and Tables

**Table 1:** Recent photosensitizers loaded or conjugated with nanocarriers for application in phototherapy.

Types of Nanocarriers	Therapeutic Modalities	PDT Agents	PTT Agents	Imaging Modalities	Year	Ref.
Polymeric micelles	PDT	mTHPC		FL	2020	[57]
Polymeric micelles	PDT + PTT	IGG	IGG	FL	2020	[59]
Polymeric micelles	PDT	Silicon phthalocyanine			2020	[60]
Polymeric micelles	PDT + PTT	ICG	ICG	FL, SPECT, PA, thermal	2020	[61]
Liposomes	PDT	curcumin			2020	[64]
Liposomes	PDT	verteporfin		FL	2020	[65]
Liposomes	PDT + PTT	Ce6	Cypate	FL	2020	[66]
Liposomes	PTT + Chemo		ZnPc(PEG)_4_	FL	2020	[67]
Polymeric nanocapsules	PDT	PpIX, hypericin		FL	2018	[70]
Polymeric nanocapsules	PDT	anthraquinone			2020	[72]
Polymeric nanocapsules	PDT	Rose Bengal		FL	2020	[73]
Polymeric nanocapsules	PDT + Chemo	verteporfin		FL	2019	[74]
Carbon nanosheet	PDT + SDT	Ce6		FL	2020	[78]
Carbon dot	PDT + Chemo	Ce6		FL	2020	[79]
Graphene oxide nanosheet	PDT	Ce6		FL	2020	[80]
Gold nanocluster	PDT + Chemo	PpIX			2020	[84]
Gold nanorod	PDT + PTT	Ce6		FL	2020	[85]
Gold nanorod	PDT	TMPy		FL	2020	[86]
Magnetic NP	PDT	MB		FL	2020	[88]
Magnetic NP	PTT		ICG	MR/US/FL	2020	[90]
Upconversion NP	PDT	pheophorbide		FL	2020	[94]
Upconversion NP	PDT	Ce6/Rose Bengal		FL	2020	[95]

Abbreviations: PDT: photodynamic therapy; PTT: photothermal therapy; FL: fluorescence; mTHPC: 5,10,15,20-tetrakis(m-hydroxyphenyl)chlorin; ICG: indocyanine green; SPECT: single-photon emission computed tomography; PA: photoacoustic; Ce6: chlorin e6; SDT: sonodynamic therapy; PpIX: protoporphyrin IX; NP: nanoparticle; MB: methylene blue; PpIX: protoporphyrin IX; TMPy: 5,10,15,20-tetrakis(1-methyl 4-pyridinio)porphyrin tetra(*p*-toluenesulfonate).

**Table 2:** Clinical trials of PDT in non-oncologic diseases.

Disease	Photo-Sensitizer	Light Source	Wave-Length (nm)	Power Density (mW/cm^2^)	Energy Density (J/cm^2^)	Treatment Protocol	Outcome of PDT Group	Enrolled Patients	Ref.
Acne	5-ALA	LED	633	100	50	20 min	significantly improved acne lesion and reduced scar formation	75	[101]
Warts	5-ALA	Halogen lamp	590–700	50	70	23 min 20 s	reduced area and number of warts than placebo group	45	[104]
Photoaging	5-ALA	Xenon lamp	400–720	3500	10.5	3 s 3 times	better wrinkle reduction than ND:YAG laser therapy group	13	[108]
Cutaneous leishmaniasis	5-ALA	LED	633		100	Once a week for 4 weeks	better treatment outcome than control group	57	[119]
Onychomycosis	MAL	LED	635		37	Once a week for 3 weeks	better clinical response than placebo group, but failed statistical significance	40	[123]
Central serous chorioretinopathy	Verteporfin	Laser	689		50	83 s	better clinical response than high-density subthreshold micropulse laser treatment group	179	[139]
Age-related macular degeneration	Verteporfin	Laser	689	600	50	83 s	additional therapeutic effect with anti-VEGF therapy	322	[143]
Corneal neo-vascularization	Verteporfin	Laser	689	600	50	83 s	combination with anti-VEGF therapy showed best therapeutic response	7	[147]
Esophageal varix	HMME	Laser		150		40 min	less newly visible vessel than control group	14	[155]
Periodontitis	ICG	laser	810	200		30 s	additional therapeutic effect with scaling and root planing	29	[160]

Abbreviations: PDT: photodynamic therapy; 5-ALA: 5-aminolevulinic acid; MAL: methyl-aminolevulinate; HMME: hematoporphyrin monomethyl ether; ICG: indocyanine green, LED: light-emitting diode; anti-VEGF: antivascular endothelial growth factor 4. Non-oncologic applications of photothermal therapy.

## Data Availability

Data sharing not applicable.
